# Scoping Review of Published Oncology Meta-analyses in High-Impact Oncology Journals

**DOI:** 10.1001/jamanetworkopen.2023.18877

**Published:** 2023-06-26

**Authors:** Alyson Haslam, Jordan Tuia, Vinay Prasad

**Affiliations:** 1 School of Medicine, University of California, San Francisco

## Abstract

**Importance:**

Many meta-analyses have been conducted on a wide array of topics, and many of these have focused on treatment efficacy of drugs or bias in interventional studies on a specific topic.

**Objective:**

To examine the factors associated with having a positive study conclusion in meta-analyses in the field of oncology.

**Evidence Review:**

All meta-analyses published between January 1, 2018, and December 31, 2021, on 5 oncology journal websites were identified and study characteristics, study results, and information on study authors were abstracted. The meta-analysis authors’ conclusions were coded as positive, negative, or equivocal, and each article subject matter was coded as one that could affect profits and marketing of a company. Whether an association existed between study characteristics and authors’ conclusions was also examined.

**Findings:**

Database searches resulted in 3947 potential articles, of which 93 meta-analyses were included in this study. Of the 21 studies with author funding from industry, 17 studies (81.0%) reported favorable conclusions. Of the 9 studies that received industry funding, 7 (77.8%) reported favorable conclusions, and of the 63 studies that did not have author or study funding from industry, 30 (47.6%) reported favorable conclusions. Studies that were funded through nonindustry sources and authors who had no relevant conflict of interest had the lowest percentage of positive conclusions and the highest percentage of negative and equivocal conclusions compared with studies with other sources of potential conflict of interest.

**Conclusions and Relevance:**

In this cross-sectional study of meta-analyses published in oncology journals, multiple factors were associated with having a positive study conclusion, which suggests that future research should be performed to elucidate reasons for more favorable conclusions among studies with study or author industry funding.

## Key Points

**Question:**

What are the characteristics of meta-analyses published in oncology journals, and what are the study characteristics associated with favorable outcomes?

**Findings:**

This scoping review of 93 meta-analyses found that many meta-analyses report on a drug, include randomized studies, and often have an author funded by industry. Methodologic quality was no different between studies with study or author industry funding and those with independent research, but industry funding was associated with the tone of the authors’ conclusion.

**Meaning:**

The multiple factors associated with having a positive study conclusion suggest that future research should be performed to elucidate reasons for more favorable conclusions among studies with study or author industry funding.

## Teaser

This scoping review examines meta-analyses published in oncology journals to identify the factors associated with having a positive study conclusion.

## Introduction

The meta-analysis is an increasingly used study design that allows results of multiple studies to be pooled to find an overall estimate.^1^ Meta-analysis has been traditionally viewed as the strongest form of evidence because of its ability to generate a pooled estimate based on the totality of evidence.^2^ However, there are certain times when a meta-analysis is appropriate and when it is not, and prior work has shown that meta-analyses and very large randomized clinical trials can reach divergent estimates.^3,4^ For example, a meta-analysis should have clinically appropriate and meaningful pooling based on interventions and outcomes.

Furthermore, biases in the individually included studies and in the conduct of a meta-analysis may result in biased pooled results that limit reliability and validity. When meta-analyses are conducted, authors should be aware of potential biases. For example, bias can occur when the meta-analysis is authored by people who are experts in the topic area and who have authored studies that could be included in the review, thus resulting in a situation that promotes their work or because of findings that are in alignment with specialty-specific recommendations.^5^ Biases can result because of the sample sizes of the included studies, with small studies often reporting larger effect sizes than larger studies.^6^ This occurrence relates to heterogeneity, which can bias results if not properly handled in the review or meta-analysis.^7^

Preregistration of a meta-analysis protocol may help reduce bias occurring from post hoc decision-making and provide greater transparency in study methods, and the use of multiple databases can help to identify all available evidence rather than a possibly biased selection.^1^ Many meta-analyses have been conducted on a wide array of topics. In the medical specialty of oncology, many of these meta-analyses have focused on treatment efficacy of drugs or bias in interventional studies on a specific topic.^8,9,10^ To our knowledge, there have been no scoping analyses evaluating the general characteristics of meta-analyses in oncology journals and factors associated with bias. Therefore, we performed a scoping review to examine the factors associated with having a positive study conclusion in meta-analyses in the field of oncology.

## Methods

We sought to systematically assemble a list of oncology meta-analyses by searching common oncology journal websites for all meta-analyses published between January 1, 2018, and December 31, 2021. In accordance with 45 CFR §46.102(f), this scoping review was not submitted for University of California, San Francisco Institutional Review Board approval because it involved publicly available data and did not involve individual patient data. We followed the Strengthening the Reporting of Observational Studies in Epidemiology (STROBE) reporting guidelines.^11^

We selected *JAMA Oncology*, *Journal of Clinical Oncology*, *Annals of Oncology*, *Lancet Oncology*, and *Clinical Cancer Research* because these journals had the 5 highest impact factors (per Scimago Journal & Country Rank) among oncology journals that publish meta-analyses. We searched each journal’s website by using the term *meta-analysis* in the search bar, allowing search results from all oncology journals affiliated with the parent journal, and limiting to the predefined dates. When there was the option, we also limited the search to research and review articles. For this review, we did not include studies that were systematic reviews only. Because we were assessing the general landscape of meta-analyses in oncology, we included all studies that had a meta-analysis design, as described by the author of the meta-analysis, and we had no further inclusion or exclusion criteria.

For all studies, we abstracted data on journal, year of publication, intervention type, tumor type, outcome type, data source, years of included trials, number of studies, total number of participants, study design included, main outcome, whether the pooled results were calculated with random or fixed effects or both, rationale explained for type of modeling used (random vs fixed), *I*^2^ for the primary outcome (or first primary outcome mentioned), heterogeneity categorization, whether the meta-analysis had been preregistered, whether an assessment of study quality or bias was performed, tool used for study quality or bias assessment, whether the data were dual reviewed, source of study funding, author conflict of interest, country of first author, and names of first and last authors. Outcome type was grouped into overall survival, tumor response (progression-free survival and response rate), safety and adverse events, behavior, fertility, pain, risk, testing, or other. For years of included trials, if the meta-analysis included studies from the inception of PubMed or MEDLINE, we determined that this was from 1966. For heterogeneity categorization, we defined an *I*^2^ of less than 25% as low, 25% to 49% as low to moderate, 50% to 74% as moderate to high, and 75% or above as high. For each first and last author, we searched PubMed for the number of meta-analyses on which the individual was a coauthor. If we were unable to find the specific author, we searched Google Scholar for data on the number of coauthored meta-analyses. We only used Google Scholar data if there was a user profile with a verified affiliation. We then categorized the number of author publications into less than 10, 10 to 24, and greater than 24. However, for logistic regression, this was used as a continuous variable.

We coded the overall tone of the authors’ conclusion, based on the abstract and at the end of the discussion, as positive, negative, or equivocal. We coded a study as positive if the authors’ conclusion promoted the intervention or exposure being studied. We coded a study as negative if the authors’ conclusion was negative toward an intervention or exposure (eg, high adverse events among the exposed). We coded a study as equivocal if we were unable to determine whether the authors’ conclusion supported or refuted an intervention or exposure. Examples of how studies were coded are provided in eTable 1 in Supplement 1. The authors’ conclusion was double coded (A.H. and J.T.), discrepancies were discussed, and a third person (V.P.) adjudicated.

We evaluated potential conflicts of interest in 2 ways. First, we categorized funding generally—whether the study or author received any funding from industry or not. These variables were coded as industry, nonindustry, none, or not reported. Second, we coded each article subject matter as one that could affect profits and marketing of a company (eg, efficacy and safety of a drug or device or prevalence of a targetable biomarker) or not (eg, association of smoking or sex with risk of disease). We then coded each study as having potential conflicts of interest for the author (eg, author received money from a company that could benefit from study findings), study (eg, study was funded by a company that could benefit from study findings), or independent (eg, no author or study payments from a company that could benefit from study findings). If a study had industry funding, we assumed that at least 1 of the authors had industry funding to conduct the study. If there was no study funding, we then looked to see if the authors reported receipt of funding from industry. Information on conflicts of interest was only obtained from information reported in the published meta-analysis.

We calculated numbers (percentages) or medians (IQRs) for characteristics overall and stratified results by type of meta-analysis. We used a Fisher exact test to examine the difference in the type of potential conflicts of interest and the number of studies with positive, negative, or equivocal conclusions. We used logistic regression to examine factors associated with a positive conclusion or not. For the regression model, we included variables on study (year of publication, number of included study participants, intervention marketable by funder, registration, heterogeneity, assessment of study quality, dual review, country of authors, study design of included studies, and number of years of included studies) and author characteristics (number of published meta-analyses by first and last author) and manually removed variables one at a time if their removal resulted in a lower Akaike information criterion value. We used a 2-sided α = .05 for statistical significance. The analyses were performed in R statistical software, version 4.2.1 (R Foundation for Statistical Computing).

## Results

**Figure 1.**
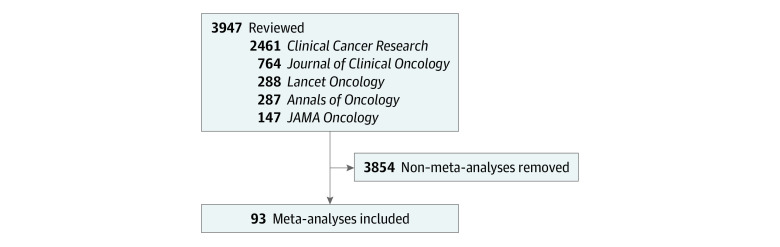
Search Results for Oncology Meta-analyses in Top Oncology Journals

Our searches resulted in 3947 potential articles (Figure 1), of which 93 meta-analyses were included in our analysis (eTable 3 in Supplement 1). Results, stratified by type of meta-analysis, are presented in Table 1. Most meta-analyses were published in the *Journal of Clinical Oncology* (31 [33.3%]) or *JAMA Oncology* (28 [30.1%]). The year with the greatest number of meta-analyses published was 2020 (28 [30.1%]). The median number of years included in a meta-analysis was 51 (IQR, 19-53). The median number of included studies was 23 (IQR, 10-42), and the median number of participants was 7584 (IQR, 2187-21 402).

**Table 1.** Characteristics of Meta-analyses Published in Oncology Journals, 2018-2021

Characteristic	No. (%)			
	Aggregate study level (n = 65)	Network (n = 9)	Individual patient level (n = 19)	Overall (N = 93)
Year				
2018	16 (24.6)	2 (22.2)	3 (15.8)	21 (22.6)
2019	15 (23.1)	2 (22.2)	5 (26.3)	22 (23.7)
2020	18 (27.7)	4 (44.4)	6 (31.6)	28 (30.1)
2021	16 (24.6)	1 (11.1)	5 (26.3)	22 (23.7)
Journal				
* Annals of Oncology*	13 (20.0)	1 (11.1)	3 (15.8)	17 (18.3)
* Clinical Cancer Research*	3 (4.6)	1 (11.1)	1 (5.3)	5 (5.4)
* JAMA Oncology*	22 (33.8)	3 (33.3)	3 (15.8)	28 (30.1)
* Journal of Clinical Oncology*	20 (30.8)	2 (22.2)	9 (47.4)	31 (33.3)
* Lancet Oncology*	7 (10.8)	2 (22.2)	3 (15.8)	12 (12.9)
Duration of included studies, median (IQR), y (n = 7 missing)	51 (19-53)	43 (28-52)	50 (16-53)	51 (19-53)
No. of included studies, median (IQR) (n = 2 missing)	26 (14-52)	12 (11-81)	6 (4-16)	23 (10-42)
No. of participants, median (IQR) (n = 3 missing)	9499 (3166-22 762)	6204 (5073-10 003)	1626 (883-7112)	7584 (2187-21 402)
Tumor type				
Breast	10 (15.4)	1 (11.1)	1 (5.3)	12 (12.9)
Gastroesophageal	3 (4.6)	1 (11.1)	3 (15.8)	7 (7.5)
Lung	3 (4.6)	0	0	3 (3.2)
Melanoma	2 (3.1)	0	1 (5.3)	3 (3.2)
Multiple	29 (44.6)	1 (11.1)	6 (31.6)	36 (38.7)
Myeloma	2 (3.1)	1 (11.1)	0	3 (3.2)
Prostate	5 (7.7)	2 (22.2)	3 (15.8)	10 (10.8)
Another single site	11 (16.9)	3 (33.3)	5 (26.3)	19 (20.4)
Intervention type				
Algorithm	0	0	1 (5.3)	1 (1.1)
Biomarker	8 (12.3)	0	6 (31.6)	14 (15.1)
Complementary	1 (1.5)	0	0	1 (1.1)
Drug	22 (33.8)	8 (88.9)	5 (26.3)	35 (37.6)
Epidemiology	7 (10.8)	0	2 (10.5)	9 (9.7)
Patient characteristic	11 (16.9)	0	3 (15.8)	14 (15.1)
Procedure	10 (15.4)	1 (11.1)	0	11 (11.8)
Radiation	2 (3.1)	0	2 (10.5)	4 (4.3)
Trial	4 (6.2)	0	0	4 (4.3)
Outcome type (could be >1)				
Adverse events and safety	12 (18.5)	0	2 (10.5)	14 (15.1)
Behavior	4 (6.2)	0	0	4 (4.3)
Overall survival^a^	20 (30.8)	7 (77.8)	9 (47.4)	36 (38.7)
Risk (incidence or prevalence)	15 (23.1)	0	4 (21.1)	19 (20.4)
Test	10 (15.4)	0	0	10 (10.8)
Tumor response	18 (27.7)	3 (33.3)	7 (36.8)	28 (30.1)
Other	4 (6.2)	1 (11.1)	2 (10.5)	7 (7.5)
Study design of included trials^b^				
Clinical trial (randomized and single group)	10 (15.4)	1 (11.1)	1 (5.3)	12 (12.9)
Database	0	0	4 (21.1)	4 (4.3)
Observational	24 (36.9)	0	3 (15.8)	27 (29.0)
RCT and observational	8 (12.3)	0	0	8 (8.6)
RCT only	22 (33.8)	8 (88.9)	9 (47.4)	39 (41.9)
Not indicated	1 (1.5)	0	2 (10.5)	3 (3.2)
Author funding				
Industry	43 (66.2)	7 (77.8)	13 (68.4)	63 (67.7)
Public	3 (4.6)	0	0	3 (3.2)
None	19 (29.2)	2 (22.2)	6 (31.6)	27 (29.0)
Study funding				
Industry	5 (7.7)	0	2 (10.5)	7 (7.5)
Other	24 (36.9)	4 (44.4)	10 (52.6)	38 (40.9)
Not reported	25 (38.5)	4 (44.1)	4 (21.1)	33 (35.5)
None	11 (16.9)	1 (11.1)	3 (15.8)	15 (16.1)
Country of authors				
Asia	3 (4.6)	1 (11.1)	1 (5.3)	5 (5.4)
Europe	10 (15.4)	0	2 (10.5)	12 (12.9)
US	15 (23.1)	3 (33.3)	4 (21.1)	22 (23.7)
Multiple or other	37 (56.9)	5 (55.6)	12 (63.2)	54 (58.1)
No. of meta-analyses by first author^b^				
<10	50 (76.9)	4 (44.4)	13 (68.4)	67 (72.0)
10-24	9 (13.8)	5 (55.6)	6 (31.6)	20 (21.5)
>24	4 (6.2)	0	0	4 (4.3)
Undetermined	2 (3.1)	0	0	2 (2.2)
No. of meta-analyses by last author				
<10	36 (55.4)	4 (44.4)	11 (57.9)	51 (54.8)
10-24	14 (21.5)	3 (33.3)	3 (15.8)	20 (21.5)
>24	12 (18.5)	1 (11.1)	4 (21.1)	17 (18.3)
Undetermined	3 (4.6)	1 (11.1)	1 (5.3)	5 (5.4)
Protocol registration	27 (41.5)	3 (33.3)	5 (26.3)	35 (37.6)
Dual review of coding^b^	50 (76.9)	7 (77.8)	4 (21.1)	61 (65.6)
Assessment of study quality^b^	37 (56.9)	6 (66.7)	2 (10.5)	45 (48.4)
Heterogeneity				
Low	11 (16.9)	1 (11.1)	3 (15.8)	15 (16.1)
Low to moderate	6 (9.2)	0	1 (5.3)	7 (7.5)
Moderate to high	6 (9.2)	1 (11.1)	0	7 (7.5)
High	23 (35.4)	2 (22.2)	2 (10.5)	27 (29.0)
Variable	4 (6.2)	1 (11.1)	1 (5.3)	6 (6.5)
Not indicated	15 (23.1)	4 (44.4)	4 (44.4)	31 (33.3)
Random or fixed effects				
Random	41 (63.1)	5 (55.6)	6 (31.6)	52 (55.9)
Fixed	5 (7.7)	2 (22.2)	5 (26.3)	12 (12.9)
Both	11 (16.9)	1 (11.1)	3 (15.8)	15 (16.1)
Neither or not indicated	8 (12.3)	1 (11.1)	5 (26.3)	14 (15.1)
Justification for random or fixed effects	28 (43.1)	3 (33.3)	4 (21.1)	35 (37.6)

Abbreviation: RCT, randomized clinical trial.

^a^ *P* < .05.

^b^ *P* < .001 among meta-analysis type (study, participant, and network).

The most common tumor type was multiple (36 [38.7%]), but the most common single tumor type was breast (12 [12.9%]). The most common intervention type was drug (35 [37.6%]), and the most common outcome type was overall survival, with or without another outcome (36 [38.7%]). The most common type of study design was randomized clinical trials only (39 [41.9%]), and the most common analysis type was a random-effects model (52 [55.9%]). The number of studies that provided justification for using a random- or fixed-effects model was 35 (37.6%). Most studies were study-level (aggregate) meta-analyses (65 [69.9%]).

Heterogeneity was high (*I*^2^ >75%) in 27 studies (29.0%), moderate to high in 7 (7.5%), low to moderate in 7 (7.5%), low in 15 (16.1%), and not indicated in 31 (33.3%). Only 35 studies (37.6%) were preregistered before being conducting, and 45 (48.4%) evaluated the quality of included studies. Sixty-one studies (65.6%) had dual review of the abstracted data.

**Figure 2.**
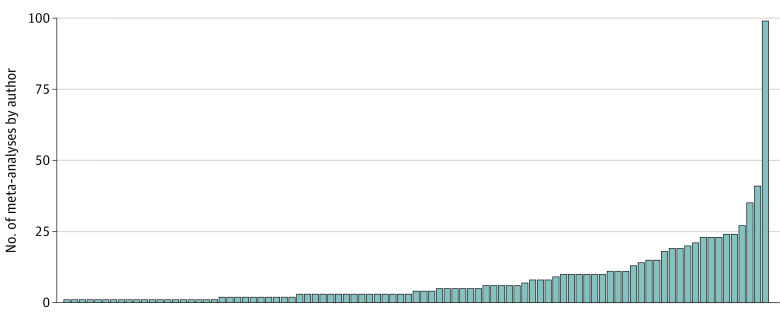
Total Number of Published Meta-analyses per Last Author of Included Meta-analyses in Oncology Journals Each bar represents a unique study.

The most common funding source was a nonindustry source (38 [40.9%]), but many did not disclose funding (33 [35.5%]). Sixty-three studies (67.7%) had authors who reported industry conflict of interest. The US was the most commonly represented geographic area (22 [23.7%]), but 54 studies (58.1%) had authors from multiple countries. Sixty-seven (72.0%) of the first authors and 51 (54.8%) of the last authors had published fewer than 10 previous meta-analyses, but it was also common for last authors to publish 25 or more meta-analyses (17 [18.3%]). Figure 2 shows the total number of published meta-analyses per last author of included meta-analyses in top oncology journals.

**Figure 3.**
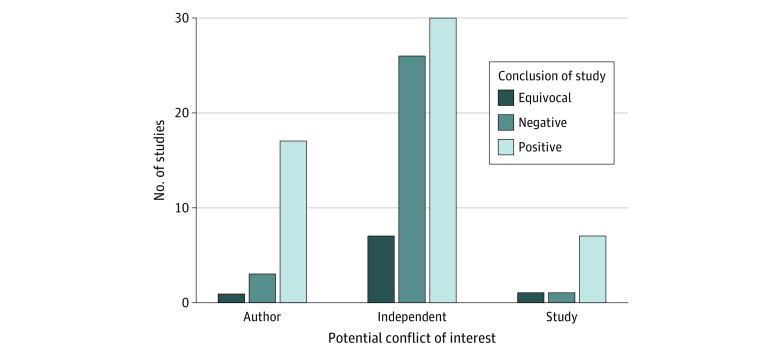
Number of Meta-analyses Reporting Positive, Negative, or Equivocal Outcomes by Potential Conflicts of Interest Author indicates received money from a company that could benefit from study findings. Study indicates study was funded by a company that could benefit from study findings. Independent indicates neither author nor study received payments or funding from a company that could benefit from study findings.

The κ statistic for study conclusion was 0.59, indicating moderate agreement. Of the 21 studies with author funding from industry, 17 studies (81.0%) reported favorable conclusions. Of the 9 studies that received industry funding, 7 (77.8%) reported favorable conclusions, and of the 63 studies that did not have author or study funding from industry, 30 (47.6%) reported favorable conclusions (Figure 3). Studies that were funded through nonindustry sources and studies with authors who had no relevant conflicts of interest had the lowest percentage of positive conclusions and the highest percentage of negative and equivocal conclusions compared with studies with other sources of potential conflicts of interest.

In the adjusted logistic analyses (Table 2), having author or study funding from a company with ties to the intervention being tested (odds ratio, 3.93; 95% CI, 1.27-13.71) was associated with higher odds of a favorable study finding compared with studies that had no author or study funding. Having a first author with a higher number of meta-analysis publications was associated with a higher likelihood (odds ratio, 1.09; 95% CI, 1.01-1.20) of reporting a favorable study finding. We found no differences in measures of study quality (eg, protocol registration, study quality assessment, dual review of data, and heterogeneity) among studies funded by industry, authors receiving industry funding, or independently funded or nonfunded research (eTable 2 in Supplement 1).

**Table 2.** Logistic Regression Testing Factors Associated With Study Conclusions (Positive vs Negative or Equivocal) in Published Oncology Meta-analyses, 2018-2021

Characteristic	Estimate	Odds ratio (95% CI)
Potential conflicts of interest related to the topic of the study		
Independent^a^	1 [Reference]	1 [Reference]
Study or author^a^	1.37	3.93 (1.27-13.71)
No. of prior meta-analyses by first author	0.09	1.09 (1.01-1.20)
No. of prior meta-analyses by last author	−0.01	0.99 (0.96-1.01)
Total No. of included patients in trial		
<2000	1 [Reference]	1 [Reference]
2001-10 000	−0.79	0.45 (0.10-1.77)
10 001-25 000	−0.85	0.43 (0.07-2.34)
>25 000	−0.82	0.44 (0.08-2.26)
Year of study publication		
2018	1 [Reference]	1 [Reference]
2019	1.75	5.76 (1.24-31.88)
2020	0.78	2.19 (0.54-9.59)
2021	0.67	1.96 (0.45-9.04)

^a^ Independent of conflicts of interest was considered if neither author nor study received payments or funding from a company that could benefit from the study findings. Study or author conflicts of interest was considered if either received payments or funding from a company that could benefit from the study findings.

## Discussion

In this scoping review, we found that for meta-analyses in top oncology journals, drug interventions were the most studied intervention type, but biomarkers and patient characteristics were also commonly studied intervention types. This study also found that intervention-related industry payments to authors or study sponsorship were associated with a higher probability of a meta-analysis finding favorable results for an intervention, when compared with non–industry-funded studies. These reports are consistent with results from a prior study^12^ exploring the role of financial conflict of interest on the conclusions of cost-effectiveness research.

Potential conflicts of interest are well recognized for being a factor for biasing study results.^13^ Although it is often accepted that registration trials are funded by and written by employees of industry, meta-research, including meta-analyses and cost-effectiveness studies that inform health policy, may be more susceptible to industry influence (either by choice of topic or by methodologic choices within topics). Moreover, a prior Cochrane review^14^ found that conflicts of interest are common and associated with favorable conclusions for general medicine drug and device studies. A previous report^12^ found that meta-analyses on cost-effectiveness studies in oncology resulted in a 40-fold greater likelihood of concluding that a drug is cost-effective. However, potential conflicts of interest are often not reported in general or oncology-specific meta-analyses.^15^

Although there are several possible explanations for why industry-funded studies are more often positive, one may be that industry is incentivized to focus on avenues of research that they know will be successful. Conversely, industry-funded studies may also rely more on surrogate end points^16^ rather than important clinical end points, which may be more likely to be positive. Another explanation is that in contrast with randomized clinical trials with prespecified statistical plans, cost-effectiveness research and meta-analyses have more analytic flexibility. Indeed, analytic choices can lead to widely divergent outcome estimates.^17^

We found that many meta-analyses were traditional meta-analysis, with a study as the unit of observation, but one-third are other types. Network meta-analyses are becoming popular because they allow researchers to compare effectiveness of multiple interventions. Still, approximately 20% of meta-analyses used patient-level data. Besides the obvious deciding factor of whether the researcher has access to individual-level data, the advantages and disadvantages of each type of meta-analysis has led to debate about which one is better, although patient-level meta-analysis may be more preferred when determining efficacy.^18,19^

We found that most meta-analyses had high heterogeneity in their pooled analysis, yet, although most studies used a random-effects model, there was no justification provided for using this method of analysis. Some authors have suggested that even with the use of a random-effects model, if there is a high degree of heterogeneity, the results should not be pooled.^20^ However, if there was no heterogeneity in study results, there would be little reason to conduct a meta-analysis because the answer would be known. An advantage of a patient-level meta-analysis is the ability to investigate heterogeneity due to individual-level effect modifiers and not only study-level factors or ecologic summaries of individual-level factors (eg, mean age of the sample). To balance the expectation of equipoise and conducting appropriate tests that accommodate heterogeneity in study results, researchers have suggested alternative methods of statistical analysis^21^ and ways in which to present the data, such as using prediction intervals in addition to the traditional *I*^2^ value.^22^ Although we did not perform an exhaustive review of reporting quality, we found that, for the items we evaluated, the quality of reporting in studies was moderate at best, with most studies (65.6%) having dual review of study data, but fewer studies reporting the quality of included studies (48.4%), providing a rationale for type of model used (37.6%%), or preregistering their study (37.6%).

### Limitations

Our analysis was limited by several factors. First, we included a search of only 5 oncology journal websites over the course of 4 years, and our results may not be applicable to the meta-analysis literature at large. These journals may have a better editorial process than other journals often selecting higher quality manuscripts and the results are likely a better-case scenario because of better study methods. Second, our analysis was purely descriptive, and we did not examine factors associated with our findings. Third, we made several assumptions with the years of included studies (eg, studies published since 1966), so our years of study inclusion may be overestimated. Fourth, we were not able to determine numbers of meta-analyses that some authors had published, so data are limited for those variables.

## Conclusions

In this scoping review of meta-analyses in oncology journals, we found that most studies either did not report on heterogeneity or had a high degree of heterogeneity, and most studies used a random-effects model but did not provide justification for its use. We also found that study funding or author conflicts of interest and having a first author with more published meta-analyses were associated with more favorable conclusions. The multiple factors associated with having a positive study conclusion suggest that future research should be performed to elucidate reasons for more favorable conclusions among studies with study or author industry funding.

## Notes

Supplement 1. **eTable 1.** Example of How Authors Conclusions Were Coded in Oncology Meta-analyses Published in Top Oncology Journals (2018-2021) **eTable 2.** Study Characteristics of Oncology Meta-analyses Published in Top Oncology Journals (2018-2021), Stratified by Potential Conflict of Interest **eTable 3. **List of Included Meta-analyses

Supplement 2.
Data Sharing Statement
