# Supplementary material for: Scoping Review of Published Oncology Meta-analyses in High-Impact Oncology Journals
Source: JAMA Netw Open. 2023 Jun 26;6(6):e2318877. doi: 10.1001/jamanetworkopen.2023.18877 (PMC10293908; doi:10.1001/jamanetworkopen.2023.18877)
Supplement: Supplement 1. — eTable 1. Example of How Authors Conclusions Were Coded in Oncology Meta-analyses Published in Top Oncology Journals (2018-2021) eTable 2. Study Characteristics of Oncology Meta-analyses Published in Top Oncology Journals (2018-2021), Stratified by Potential Conflict of Interest eTable 3. List of Included Meta-analyses [file jamanetwopen-e2318877-s001.pdf]

Supplementary Online Content

Haslam A, Tuia J, Prasad V. Scoping review of published oncology meta-analyses in high-impact oncology journals. *JAMA Netw Open*. 2023;6(6):e2318877. doi:10.1001/jamanetworkopen.2023.18877

eTable 1. Example of How Authors’ Conclusions Were Coded in Oncology Meta-analyses Published in Top Oncology Journals (2018-2021)

eTable 2. Study Characteristics of Oncology Meta-analyses Published in Top Oncology Journals (2018-2021), Stratified by Potential Conflict of Interest

eTable 3. List of Included Meta-analyses

This supplementary material has been provided by the authors to give readers additional information about their work.

© 2023 Haslam A et al. *JAMA Network Open.*

eTable 1. Example of How Authors' Conclusions Were Coded in Oncology Meta-analyses Published in Top Oncology Journals (2018-2021)

Study title	Text	Coding
Therapeutic Options for Neuroendocrine Tumors: A Systematic Review and Network Meta-analysis	"There appears to be a range of efficient therapies with different safety profiles available for patients with neuroendocrine tumors."	Equivocal
Association of Patient Sex With Efficacy of Immune Checkpoint Inhibitors and Overall Survival in Advanced Cancers: A Systematic Review and Meta-analysis	"…we found no difference in immunotherapy efficacy or OS [overall survival] between women and men. Contrary to findings of a previous analysis, we found no evidence that sex should be considered when deciding whether to offer immunotherapy to patients with advanced cancers."	Equivocal
Body mass index and 20 specific cancers: re-analyses of dose–response meta-analyses of observational studies	"The association of increased BMI [body mass index] and cancer is heterogeneous across cancer types."	Equivocal
Use of Immunotherapy With Programmed Cell Death 1 vs Programmed Cell Death Ligand 1 Inhibitors in Patients With Cancer: A Systematic Review and Meta-analysis	"Our meta-analysis suggests that anti–PD-1 exhibited better survival outcomes than anti–PD-L1 in patients with solid tumors in either overall, monotherapy, or combination therapy settings, with comparable safety profiles."	Positive
Clinical Evidence for Association of Acupuncture and Acupressure With Improved Cancer Pain: A Systematic Review and Meta-Analysis	"This systematic review and meta-analysis found that acupuncture and/or acupressure was significantly associated with reduced cancer pain and decreased use of analgesics, although the evidence level was moderate."	Positive
Association of Measurable Residual Disease With Survival Outcomes in Patients With Acute Myeloid Leukemia: A Systematic Review and Meta-analysis	"…achievement of MRD [measurable residual disease] negativity is associated with superior DFS [disease-free survival] and OS [overall survival] in patients with AML [acute myeloid leukemia]."	Positive
A Systematic Review and Meta-Analysis on the Predictive Value of Cell-Free DNA–Based Androgen Receptor Copy Number Gain in Patients With Castration-Resistant Prostate Cancer	"…AR-V7 [androgen receptor splice variant 7] is a potential biomarker of treatment resistance in mCRPC [metastatic castration-resistant prostate cancer] patients and AR-V7-positive mCRPC patients had shorter OS than AR-V7-negative patients when treated with ARSi [androgen receptor signalling inhibitors]or	Negative

© 2023 Haslam A et al. *JAMA Network Open.*

	taxane. AR-V7-positive mCRPC patients showed poorer outcomes including PSA [prostate-specific antigen] response, PFS [progression-free survival], and OS than AR-V7-negative patients when receiving ARSis.”	
Fatal Toxic Effects Associated With Immune Checkpoint Inhibitors: A Systematic Review and Meta-analysis	“In the largest evaluation of fatal ICI [immune checkpoint inhibitors]-associated toxic effects published to date to our knowledge, we observed early onset of death with varied causes and frequencies depending on therapeutic regimen. Clinicians across disciplines should be aware of these uncommon lethal complications.”	Negative
Carfilzomib-Associated Cardiovascular Adverse Events: A Systematic Review and Meta-analysis	“Carfilzomib was associated with a significant incidence of CVAE [cardiovascular adverse events], with higher rates seen with higher doses of carfilzomib… Future studies are needed to identify patients at high risk for CVAE, develop optimal monitoring strategies, and explore strategies to mitigate these risks.”	Negative

© 2023 Haslam A et al. *JAMA Network Open.*

eTable 2. Study Characteristics of Oncology Meta-analyses Published in Top Oncology Journals (2018-2021), Stratified by Potential Conflict of Interest

	**Independent (n=63)**	**Study funding from industry (n=9)**	**Author funding from industry (n=21)**	**p-value**
**Year (%)**				0.90
2018	15 (23.8)	2 (22.2)	4 (19.0)	
2019	14 (22.2)	2 (22.2)	6 (28.6)	
2020	17 (27.0)	3 (33.3)	8 (38.1)	
2021	17 (27.0)	2 (22.2)	3 (14.3)	
**Meta-analysis type (%)**				0.11
Network	4 (6.3)	0 (0.0)	5 (23.8)	
Patient	12 (19.0)	3 (33.3)	4 (19.0)	
Study/aggregate	47 (74.6)	6 (66.7)	12 (57.1)	
**Geographical region (%)**				0.64
Asia	5 (7.9)	0 (0.0)	0 (0.0)	
Europe	8 (12.7)	2 (22.2)	2 (9.5)	
Multiple	37 (58.7)	5 (55.6)	12 (57.1)	
US	13 (20.6)	2 (22.2)	7 (33.3)	
**Study design (%)**				0.12
Clinical trials	8 (12.7)	1 (11.1)	3 (14.3)	
Database	3 (4.8)	0 (0.0)	1 (4.8)	
Not indicated	2 (3.2)	0 (0.0)	1 (4.8)	
observational	23 (36.5)	1 (11.1)	3 (14.3)	
RCT and observational	8 (12.7)	0 (0.0)	0 (0.0)	
RCT only	19 (30.2)	7 (77.8)	13 (61.9)	
**Journal (%)**				0.90
**Annal of Oncology**	11 (17.5)	2 (22.2)	4 (19.0)	
Clinical Cancer Research	4 (6.3)	0 (0.0)	1 (4.8)	
JAMA Oncology	17 (27.0)	3 (33.3)	8 (38.1)	
Journal of Clinical Oncology	24 (38.1)	2 (22.2)	5 (23.8)	
Lancet Oncology	7 (11.1)	2 (22.2)	3 (14.3)	
**Included years (median (IQR))**	51 (19, 53)	50 (34, 52)	52 (18, 54)	0.59
**Number of meta-analyses by first author (median (IQR))**	3 (2, 10)	4 (2, 8)	5 (2, 13)	0.78
**Number of meta-analyses by last author (median (IQR))**	9 (3, 22)	6 (3, 22)	4 (3, 14)	0.44
**Number of meta-analyses by first author (%)**				0.81
<10	32 (50.8)	5 (55.6)	14 (66.7)	

© 2023 Haslam A et al. *JAMA Network Open.*

>25	12 (19.0)	2 (22.2)	3 (14.3)	
10-24	16 (25.4)	1 (11.1)	3 (14.3)	
undetermined	3 (4.8)	1 (11.1)	1 (4.8)	
**Number of meta-analyses by first author (%)**				0.55
<10	46 (73.0)	7 (77.8)	14 (66.7)	
>25	3 (4.8)	1 (11.1)	0 (0.0)	
10-25	12 (19.0)	1 (11.1)	7 (33.3)	
Undetermined	2 (3.2)	0 (0.0)	0 (0.0)	
**Random or fixed analysis (%)**				0.90
Both	11 (17.5)	2 (22.2)	2 (9.5)	
Fixed	7 (11.1)	1 (11.1)	4 (19.0)	
Not indicated/neither	9 (14.3)	2 (22.2)	3 (14.3)	
**Random**	36 (57.1)	4 (44.4)	12 (57.1)	
**Rationale for random effect model = y (%)**	22 (34.9)	5 (55.6)	8 (38.1)	0.49
**Heterogeneity (%)**				0.30
High	20 (31.7)	4 (44.4)	3 (14.3)	
Low	9 (14.3)	0 (0.0)	6 (28.6)	
Low/moderate	4 (6.3)	0 (0.0)	3 (14.3)	
Moderate/high	6 (9.5)	0 (0.0)	1 (4.8)	
Not indicated	19 (30.2)	5 (55.6)	7 (33.3)	
Variable	5 (7.9)	0 (0.0)	1 (4.8)	
**Protocol registration = yes (%)**	24 (38.1)	3 (33.3)	8 (38.1)	0.96
**Study quality assessment = yes (%)**	33 (52.4)	2 (22.2)	10 (47.6)	0.16
**Dual review of data = yes (%)**	43 (68.3)	4 (44.4)	14 (66.7)	0.37
**Number of included studies (median (IQR))**	26 (10, 46)	8 (6, 17)	18 (12, 42)	0.21
**Number of patients (median (IQR))**	9751 (3222, 26429)	2081 (1111, 8135)	6204 (2528, 11379)	0.69
**Conclusion (%)**				0.04
Equivocal	7 (11.1)	1 (11.1)	1 (4.8)	
Negative	26 (41.3)	1 (11.1)	3 (14.3)	
Positive	30 (47.6)	7 (77.8)	17 (81.0)	
**Marketable intervention by funder = yes (%)**	27 (42.9)	9 (100.0)	19 (90.5)	<0.001

© 2023 Haslam A et al. *JAMA Network Open.*

eTable 3. List of Included Oncology Meta-analyses

Title	Year	Author
Association of Measurable Residual Disease With Survival Outcomes in Patients With Acute Myeloid Leukemia: A Systematic Review and Meta-analysis	2020	Short et al.
Carfilzomib-Associated Cardiovascular Adverse Events: A Systematic Review and Meta-analysis	2018	Waxman et al.
Incidence of Endocrine Dysfunction Following the Use of Different Immune Checkpoint Inhibitor Regimens: A Systematic Review and Meta-analysis	2018	Barroso-Sousa et al.
Meta-analysis in metastatic uveal melanoma to determine progression free and overall survival benchmarks: an international rare cancers initiative (IRCI) ocular melanoma study	2019	Khoja et al.
A meta-analysis of reversion mutations in BRCA genes identifies signatures of DNA end-joining repair mechanisms driving therapy resistance	2021	Tobalina et al.
A meta-analysis of the accuracy of a neuroendocrine tumor mRNA genomic biomarker (NETest) in blood	2020	Öberg et al.
Ultrasound for Breast Cancer Detection Globally: A Systematic Review and Meta-Analysis	2019	Sood et al.
Meta-Analysis of Prevalence of Triple-Negative Breast Cancer and Its Clinical Features at Incidence in Indian Patients With Breast Cancer	2020	Kulkarni et al.
Revisiting Risk and Benefit in Early Oncology Trials in the Era of Precision Medicine: A Systematic Review and Meta-Analysis of Phase I Trials of Targeted Single-Agent Anticancer Therapies	2021	Mackley et al.
Efficacy and Safety of Stereotactic Radiosurgery for Brainstem Metastases: A Systematic Review and Meta-analysis	2021	Chen et al.
Comparison of Biomarker Modalities for Predicting Response to PD-1/PD-L1 Checkpoint Blockade: A Systematic Review and Meta-analysis	2019	Lu et al.
Autologous Transplantation for Newly Diagnosed Multiple Myeloma in the Era of Novel Agent Induction: A Systematic Review and Meta-analysis	2018	Dhakal et al.
Pathologic Complete Response after Neoadjuvant Chemotherapy and Impact on Breast Cancer Recurrence and Survival: A Comprehensive Meta-analysis	2020	Spring et al.
Determining the Optimal Adjuvant Therapy for Improving Survival in Elderly Patients with Glioblastoma: A Systematic Review and Network Meta-analysis	2020	Nassiri et al.
Tumor Mutational Burden, Toxicity, and Response of Immune Checkpoint Inhibitors Targeting PD(L)1, CTLA-4, and Combination: A Meta-regression Analysis	2020	Osipov et al.
Individual Patient Data Meta-Analysis of the Value of Microsatellite Instability As a Biomarker in Gastric Cancer	2019	Pietrantonio et al.
Prostate Radiotherapy With Adjuvant Androgen Deprivation Therapy (ADT) Improves Metastasis-Free Survival Compared to Neoadjuvant ADT: An Individual Patient Meta-Analysis	2021	Spratt et al.

© 2023 Haslam A et al. *JAMA Network Open.*

Meta-Analysis of PD-L1 Expression As a Predictor of Survival After Checkpoint Blockade	2020	Arfè et al.
Prevalence of Themes Linked to Delayed Presentation of Breast Cancer in Africa: A Meta-Analysis of Patient-Reported Studies	2020	Agodirin et al
Systemic Therapy and Sequencing Options in Advanced Hepatocellular Carcinoma: A Systematic Review and Network Meta-analysis	2020	Sonbol et al.
The Role of Maintenance Strategies in Metastatic Colorectal Cancer: A Systematic Review and Network Meta-analysis of Randomized Clinical Trials	2020	Sonbol et al.
Accuracy and Methodologic Challenges of Volatile Organic Compound–Based Exhaled Breath Tests for Cancer Diagnosis: A Systematic Review and Meta-analysis	2019	Hanna et al.
Multimodal Meta-Analysis of 1,494 Hepatocellular Carcinoma Samples Reveals Significant Impact of Consensus Driver Genes on Phenotypes	2019	Chaudhary et al.
Systematic review and meta-analysis of the evidence for oral nutritional intervention on nutritional and clinical outcomes during chemo(radio)therapy: current evidence and guidance for design of future trials	2018	de van der Schueren et al.
Homologous Recombination Deficiency in Pancreatic Cancer: A Systematic Review and Prevalence Meta-Analysis	2021	Casolino et al.
Receptor-Defined Breast Cancer in Five East African Countries and Its Implications for Treatment: Systematic Review and Meta-Analysis	2021	Popli et al.
Mortality in Cancer Patients With COVID-19 Who Are Admitted to an ICU or Who Have Severe COVID-19: A Systematic Review and Meta-Analysis	2021	Nadkarni et al.
Leveraging Health Information Technology to Collect Family Cancer History: A Systematic Review and Meta-Analysis	2021	Li et al.
Gamma knife radiosurgery for uveal melanomas and metastases: a systematic review and meta-analysis	2020	Parke et al.
Treatment-Related Adverse Events of PD-1 and PD-L1 Inhibitors in Clinical Trials: A Systematic Review and Meta-analysis	2019	Wang et al.
Therapeutic Options for Neuroendocrine Tumors: A Systematic Review and Network Meta-analysis	2019	Kaderli et al.
Association of Gleason Grade With Androgen Deprivation Therapy Duration and Survival Outcomes: A Systematic Review and Patient-Level Meta-analysis	2019	Kishan et al.
Maintenance Treatment and Survival in Patients With Myeloma: A Systematic Review and Network Meta-analysis	2018	Gay et al.
Prognostic Implications of PD-L1 Expression in Breast Cancer: Systematic Review and Meta-analysis of Immunohistochemistry and Pooled Analysis of Transcriptomic Data	2019	Matikas et al.
Ramucirumab Safety in East Asian Patients: A Meta-Analysis of Six Global, Randomized, Double-Blind, Placebo-Controlled, Phase III Clinical Trials	2018	Yen et al.
Efficacy of Exercise Therapy on Cardiorespiratory Fitness in Patients With Cancer: A Systematic Review and Meta-Analysis	2018	Scott et al.

© 2023 Haslam A et al. *JAMA Network Open.*

A systematic review and meta-analysis of the 2007 WCRF/AICR score in relation to cancer-related health outcomes	2020	Solans et al.
Efficacy of adoptive therapy with tumor-infiltrating lymphocytes and recombinant interleukin-2 in advanced cutaneous melanoma: a systematic review and meta-analysis	2019	Dafni et al.
Meta-Analysis of 1,200 Transcriptomic Profiles Identifies a Prognostic Model for Pancreatic Ductal Adenocarcinoma	2019	Sandhu et al.
Comparison of Systemic Treatments for Metastatic Castration-Sensitive Prostate Cancer: A Systematic Review and Network Meta-analysis	2021	Wang et al.
Evaluation of Daratumumab for the Treatment of Multiple Myeloma in Patients With High-risk Cytogenetic Factors: A Systematic Review and Meta-analysis	2020	Smith Giri
Pregnancy After Breast Cancer: A Systematic Review and Meta-Analysis	2021	Lambertini et al.
Association of Germline BRCA Pathogenic Variants With Diminished Ovarian Reserve: A Meta-Analysis of Individual Patient-Level Data	2021	Turan et al.
A Systematic Review and Meta-Analysis on the Predictive Value of Cell-Free DNA–Based Androgen Receptor Copy Number Gain in Patients With Castration-Resistant Prostate Cancer	2020	Tolmeijer et al.
Predictive and Prognostic Properties of Human Equilibrative Nucleoside Transporter 1 Expression in Gemcitabine-Treated Pancreatobiliary Cancer: A Meta-Analysis	2019	Vo et al.
Survival After Minimally Invasive vs Open Radical Hysterectomy for Early-Stage Cervical Cancer: A Systematic Review and Meta-analysis	2020	Nitecki et al.
PARP inhibitors and newly second primary malignancies in cancer patients: a systematic review and safety meta-analysis of placebo randomized controlled trials	2021	Morice et al.
Hypofractionated radiotherapy in locally advanced bladder cancer: an individual patient data meta-analysis of the BC2001 and BCON trials	2021	Choudhury et al.
Haploidentical Stem Cell Transplantation With Posttransplant Cyclophosphamide Therapy vs Other Donor Transplantations in Adults With Hematologic Cancers: A Systematic Review and Meta-analysis	2019	Gagelmann et al.
Fatal Toxic Effects Associated With Immune Checkpoint Inhibitors: A Systematic Review and Meta-analysis	2018	Wang et al.
Benefits and Harms of Lung Cancer Screening by Chest Computed Tomography: A Systematic Review and Meta-Analysis	2021	Passiglia et al.
Intermediate clinical endpoints for surrogacy in localised prostate cancer: an aggregate meta-analysis	2021	Gharzai et al.
Adverse event profile for immunotherapy agents compared with chemotherapy in solid organ tumors: a systematic review and meta-analysis of randomized clinical trials	2020	Magee et al.
Addition of Androgen-Deprivation Therapy or Brachytherapy Boost to External Beam Radiotherapy for Localized Prostate Cancer: A Network Meta-Analysis of Randomized Trials	2020	Jackson et al.
Clinical and Molecular Characteristics Associated With Survival Among Patients Treated With Checkpoint Inhibitors for Advanced Non–Small Cell Lung Carcinoma: A Systematic Review and Meta-analysis	2018	Lee et al.

© 2023 Haslam A et al. *JAMA Network Open.*

Chemotherapy and radiotherapy in locally advanced head and neck cancer: an individual patient data network meta-analysis	2021	Petit et al.
Overall Survival in Men With Bone Metastases From Castration-Resistant Prostate Cancer Treated With Bone-Targeting Radioisotopes: A Meta-analysis of Individual Patient Data From Randomized Clinical Trials	2020	Terriss et al.
Defining the Most Appropriate Primary End Point in Phase 2 Trials of Immune Checkpoint Inhibitors for Advanced Solid Cancers: A Systematic Review and Meta-analysis	2018	Ritchie et al.
Adjuvant Tyrosine Kinase Inhibitors in Renal Cell Carcinoma: A Concluded Living Systematic Review and Meta-Analysis	2021	Riaz et al.
Depression, Anxiety, and Other Mental Disorders in Patients With Cancer in Low- and Lower-Middle–Income Countries: A Systematic Review and Meta-Analysis	2021	Walker et al.
Tumor Response End Points as Surrogates for Overall Survival in Immune Checkpoint Inhibitor Trials: A Systematic Review and Meta-Analysis	2021	Kok et al.
Physical activity in relation to risk of prostate cancer: a systematic review and meta-analysis	2018	Benke et al.
Association of Patient Sex With Efficacy of Immune Checkpoint Inhibitors and Overall Survival in Advanced Cancers: A Systematic Review and Meta-analysis	2019	Wallis et al.
Effect of Psychological Intervention on Fear of Cancer Recurrence: A Systematic Review and Meta-Analysis	2019	Tauber et al.
Gonadotropin-Releasing Hormone Agonists During Chemotherapy for Preservation of Ovarian Function and Fertility in Premenopausal Patients With Early Breast Cancer: A Systematic Review and Meta-Analysis of Individual Patient–Level Data	2018	Lambertin et al.
COVID-19 and Cancer: Lessons From a Pooled Meta-Analysis	2020	Desai et al.
Risk of gastrointestinal cancers in patients with cystic fibrosis: a systematic review and meta-analysis	2018	Yamada et al.
What is the optimal systemic treatment of men with metastatic, hormone-naive prostate cancer? A STOPCAP systematic review and network meta-analysis	2018	Vale et al.
Cancer immunotherapy efficacy and patients' sex: a systematic review and meta-analysis	2018	Conforti et al.
Platinum-based neoadjuvant chemotherapy in triple-negative breast cancer: a systematic review and meta-analysis	2018	Poggio et al.
Incidence and mortality from cervical cancer and other malignancies after treatment of cervical intraepithelial neoplasia: a systematic review and meta-analysis of the literature	2020	Kalliala et al.
Outcomes Following Immune Checkpoint Inhibitor Treatment of Patients With Microsatellite Instability-High Cancers: A Systematic Review and Meta-analysis	2020	Petrelli et al.
Endocrine treatment versus chemotherapy in postmenopausal women with hormone receptor-positive, HER2-negative, metastatic breast cancer: a systematic review and network meta-analysis	2019	Giuliano et al.

© 2023 Haslam A et al. *JAMA Network Open.*

Effect of Cancer on Clinical Outcomes of Patients With COVID-19: A Meta-Analysis of Patient Data	2020	Giannakoulis et al.
Safety and Survival Rates Associated With Ablative Stereotactic Radiotherapy for Patients With Oligometastatic Cancer: A Systematic Review and Meta-analysis	2020	Lehrer et al.
Statistical controversies in clinical research: limitations of open-label studies assessing antiangiogenic therapies with regard to evaluation of vascular adverse drug events—a meta-analysis	2018	Trone et al.
Antihypertensive treatment and risk of cancer: an individual participant data meta-analysis	2021	Copland et al.
Individual Patient Data Meta-Analysis of FOLFOXIRI Plus Bevacizumab Versus Doublets Plus Bevacizumab as Initial Therapy of Unresectable Metastatic Colorectal Cancer	2020	Cremolini et al.
Prevalence of Targetable Mutations in Black Patients With Lung Cancer: A Systematic Review and Meta-Analysis	2021	Costa et al.
Prevalence of human papillomavirus DNA and p16INK4a in penile cancer and penile intraepithelial neoplasia: a systematic review and meta-analysis	2019	Olesen et al.
Predictors of Unemployment After Breast Cancer Surgery: A Systematic Review and Meta-Analysis of Observational Studies	2018	Wang et al.
Evaluation of Reproducible Research Practices in Oncology Systematic Reviews With Meta-analyses Referenced by National Comprehensive Cancer Network Guidelines	2019	Wayant et al.
Disease-free survival as a surrogate for overall survival in patients with HER2-positive, early breast cancer in trials of adjuvant trastuzumab for up to 1 year: a systematic review and meta-analysis	2019	Saad et al.
Body mass index and 20 specific cancers: re-analyses of dose–response meta-analyses of observational studies	2018	Choi et al.
Association of Smoking Status With Recurrence, Metastasis, and Mortality Among Patients With Localized Prostate Cancer Undergoing Prostatectomy or Radiotherapy: A Systematic Review and Meta-analysis	2018	Foerster et al.
Vitamin D supplementation and total cancer incidence and mortality: a meta-analysis of randomized controlled trials	2019	Keum et al.
Aspirin and the risk of colorectal and other digestive tract cancers: an updated meta-analysis through 2019	2020	Bosetti et al.
Impact of prediagnostic smoking and smoking cessation on colorectal cancer prognosis: a meta-analysis of individual patient data from cohorts within the CHANCES consortium	2018	Ordo´nez-Mena et al.
Treatment-related adverse events of PD-1 and PD-L1 inhibitor-based combination therapies in clinical trials: a systematic review and meta-analysis	2021	Zhou et al.
Genetic risk, incident gastric cancer, and healthy lifestyle: a meta-analysis of genome-wide association studies and prospective cohort study	2020	Jin et al.
Use of Immunotherapy With Programmed Cell Death 1 vs Programmed Cell Death Ligand 1 Inhibitors in Patients With Cancer: A Systematic Review and Meta-analysis	2020	Duan et al.

© 2023 Haslam A et al. *JAMA Network Open.*

Clinical Evidence for Association of Acupuncture and Acupressure With Improved Cancer Pain: A Systematic Review and Meta-Analysis	2020	He et al.
Comparative Efficacy of Therapeutics for Chronic Cancer Pain: A Bayesian Network Meta-Analysis	2019	Huang et al.

© 2023 Haslam A et al. *JAMA Network Open.*
