# Supplementary material for: Scoping Review of Published Oncology Meta-analyses in High-Impact Oncology Journals
Source: JAMA Netw Open. 2023 Jun 26;6(6):e2318877. doi: 10.1001/jamanetworkopen.2023.18877 (PMC10293908; doi:10.1001/jamanetworkopen.2023.18877)
Supplement: Supplement 2. — Data Sharing Statement [file jamanetwopen-e2318877-s002.pdf]

Data Sharing Statement

Haslam. Scoping Review of Published Oncology Meta-analyses in High-Impact Oncology Journals. *JAMA Netw Open*. Published June 20, 2023.
doi:10.1001/jamanetworkopen.2023.18877

Data
Data available: No

Additional Information
Explanation for why data not available: Data are publicly available
